# Supplementary material for: Shift in G1-Checkpoint from ATM-Alone to a Cooperative ATM Plus ATR Regulation with Increasing Dose of Radiation
Source: Cells. 2021 Dec 27;11(1):63. doi: 10.3390/cells11010063 (PMC8750242; doi:10.3390/cells11010063)
Supplement: Supplementary file 1 [file cells-11-00063-s001.zip › cells-1474523-supplementary.pdf]

Expo, 82-6 hTert

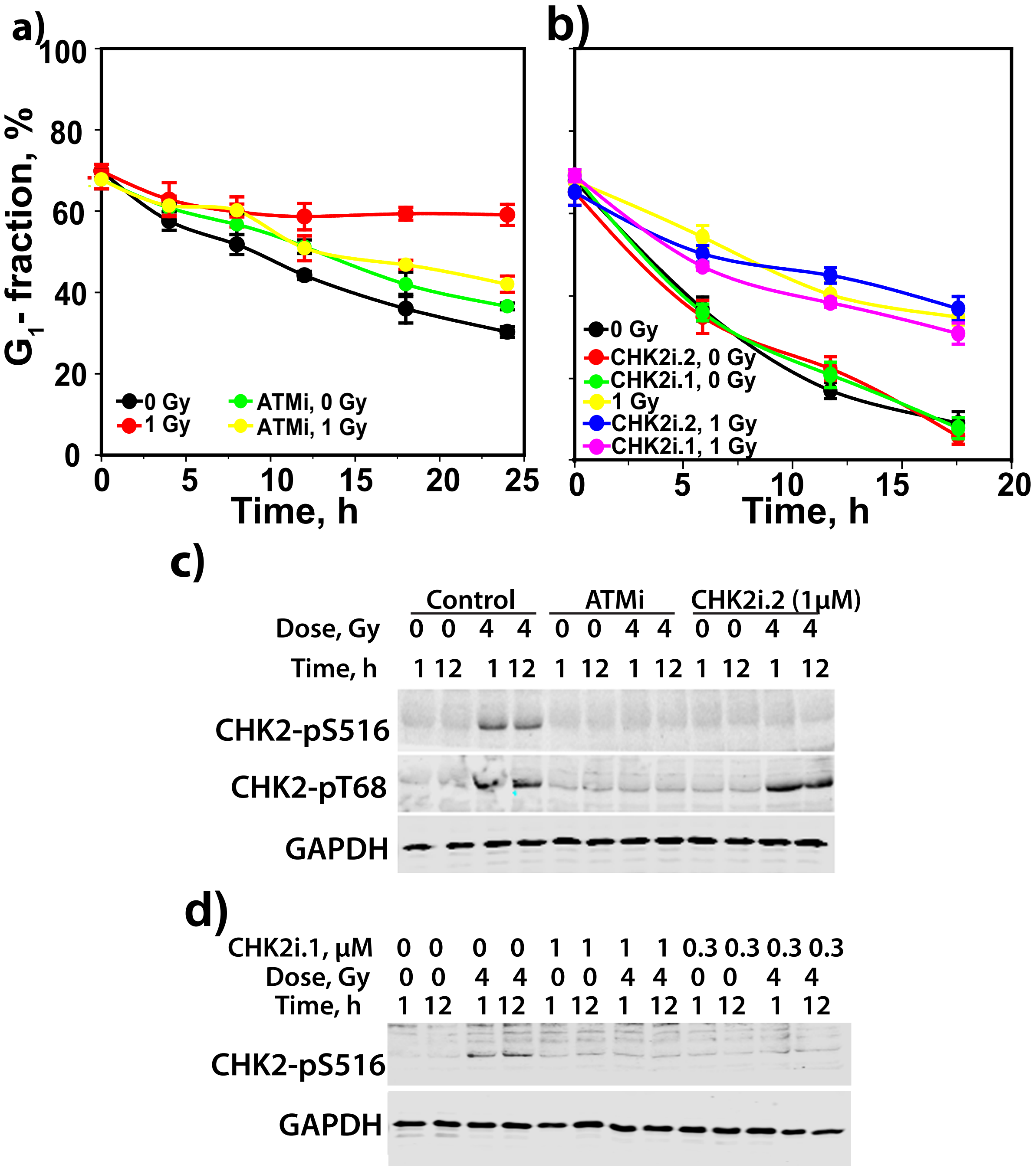

Figure S1, Li et al.

Figure S1. Demonstration of IR-induced G1-checkpoint in G1-irradiated 82-6hTert cells. a) As in Figure 1b for cells exposed to 0 or 1 Gy of X-rays in the presence or absence of ATMi as indicated. b) As in a for cells exposed to 0 or 1 Gy and the indicated inhibitors of CHK1 and CHK2. c) Effects of ATMi or CHK2i.2 on the phosphorylation of CHK2 in exponentially growing 82-6 hTert cells. d) Effects of CHK2i.1 on the phosphorylation of CHK2-S516 in exponentially growing 82-6 hTert cells. In a) and b), plotted is the mean and SE from three independent experiments. See also Table S6 for more information. In c) and d), experiments are reproduced 3 times, and a representative one is shown.

Expo, 82-6 htert

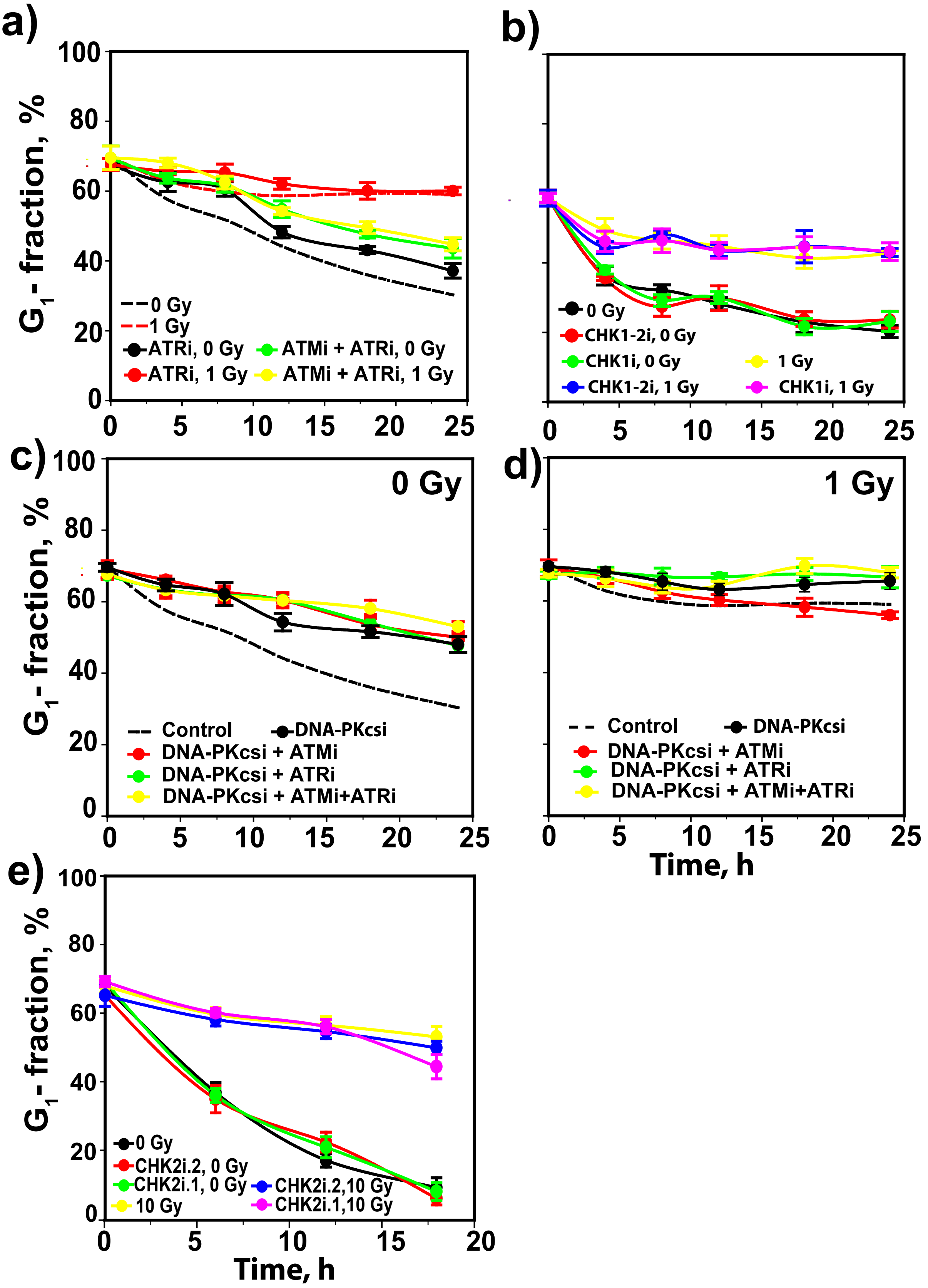

Figure S2, Li et al.

Figure S2. Effects of combinations of ATM, ATR, CHK1, CHK2 and DNA-PKcs inhibitors on low-IR-dose-induced G_1 checkpoint in G_1 irradiated 82-6 hTert cells. a) As in Figure 1b for cells exposed to 0 or 1 Gy and the indicated combinations of ATMi and ATRi. b) As in a for cells exposed to CHK1 and/or CHK2 inhibitors as indicated. c) as in a for unirradiated cells exposed to ATMi, ATRi and DNA-PKcsi as indicated. d) As in c for 1Gy irradiated cells exposed to ATMi, ATRi and DNA-PKcsi as indicated. e) As in a for cells exposed to 0 or 10 Gy and the indicated inhibitors of CHK2. Broken lines show results from Figure S1 without data point to avoid congestion. Plotted is the mean and SE from three independent experiments. See also Table S7 for more information.

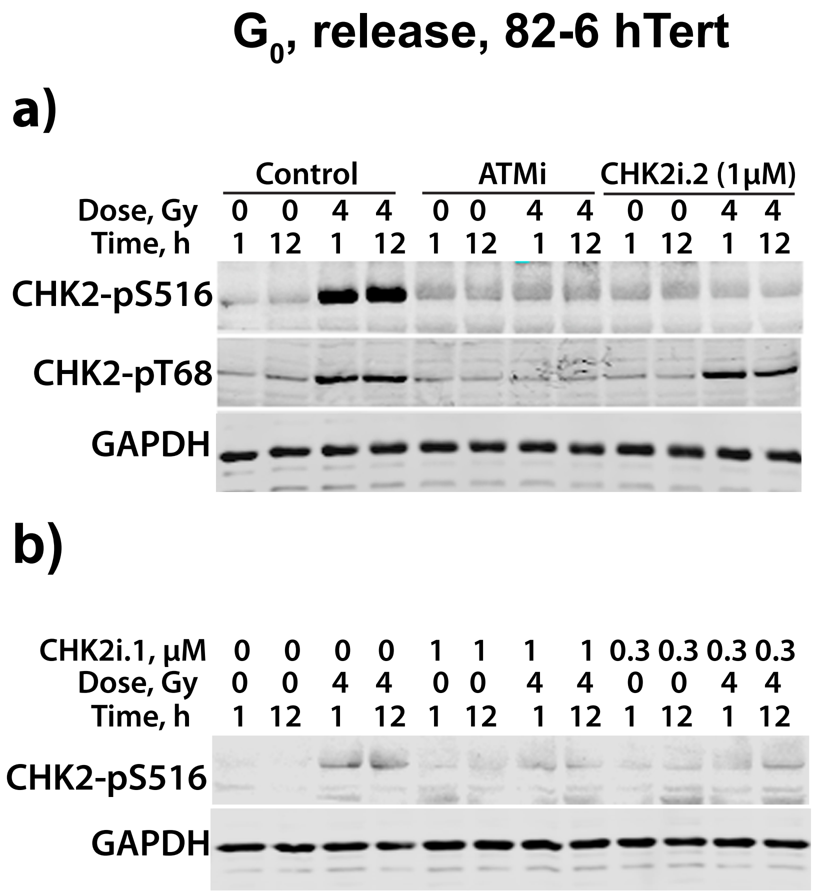

Figure S3, Li et al.

Figure S3. As in Figure S1c and S1d for G_0 cells released into the cell cycle by transfer to fresh growth medium just before irradiation and application of the indicated inhibitors. Experiments are reproduced 3 times, and a representative one is shown.

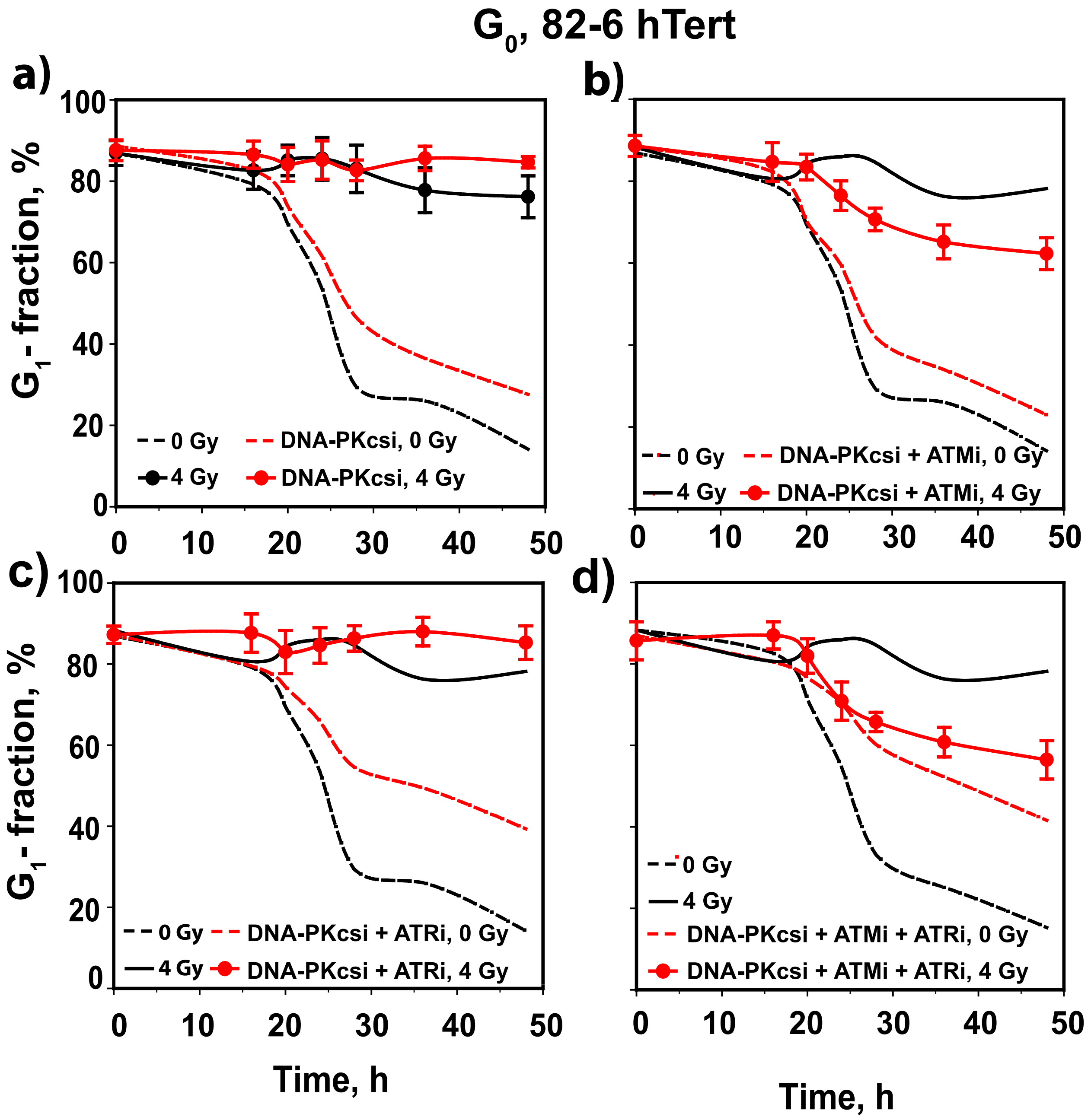

Figure S4 Li et al.

Figure S4. a) As in 1b for G_0 cells SD 82-6 hTert cells exposed to 0 or 4 Gy and treated with DNA-PKcsi as indicated. b) As in a for cells treated with combinations of ATMi and DNA-PKcsi as indicated. c) As in b for cells treated with combinations of ATRi and DNA-PKcsi inhibitors as indicated. d) As in c for cells treated with combined ATMi, ATRi and DNA-PKcsi. Lines of both broken and solid show results from Figure 4, or S4a without data point to avoid congestion. Plotted is the mean and SE from three independent experiments. See also Table S8 for more information

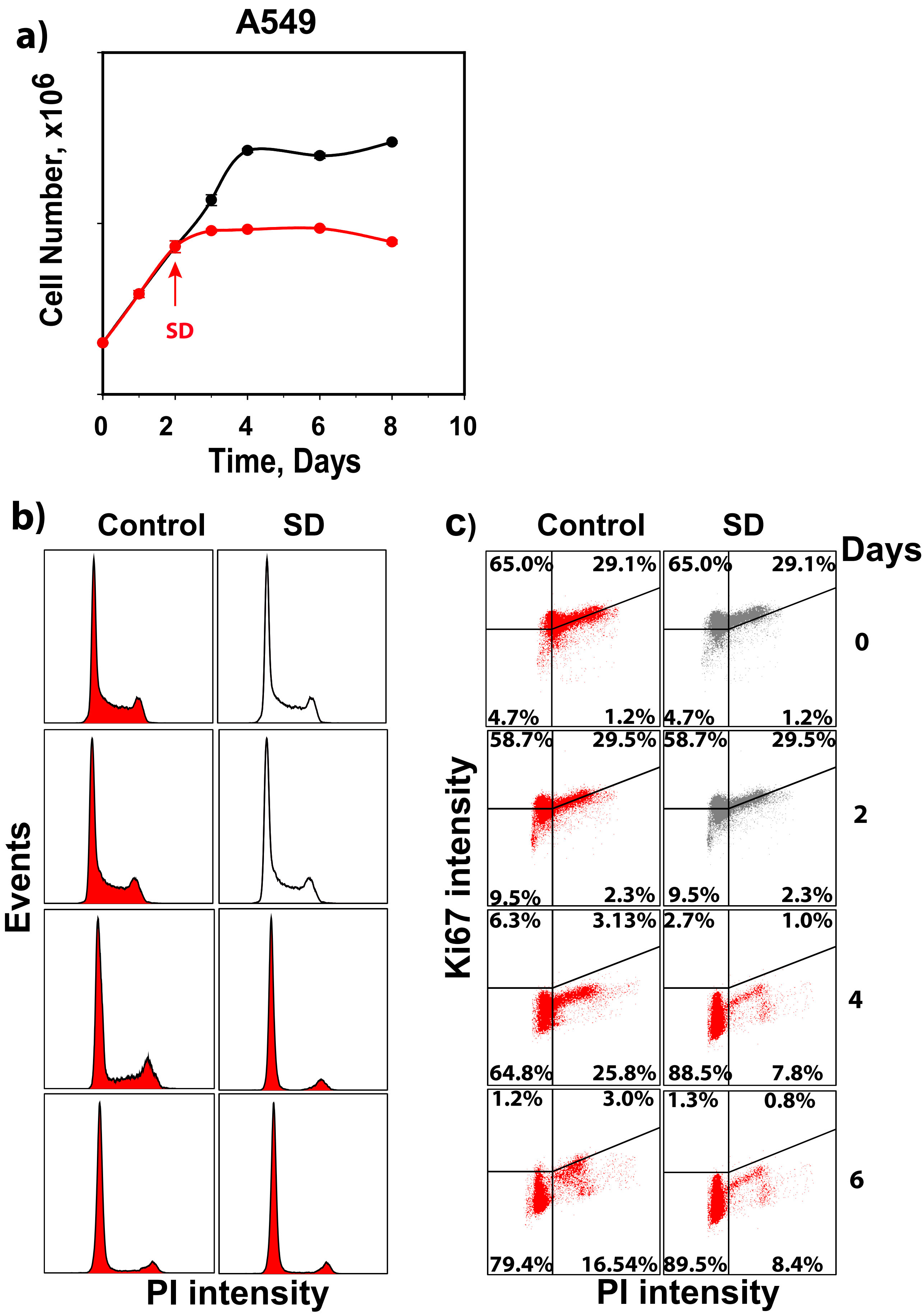

Li et al, Figure S5

Figure S5. Validation of A549 cell cultures for the analysis of IR-induced G1-checkpoint in cells irradiated in G_0. a) Proliferation of A549 cells under normal growth conditions, as well as following SD after two days. Plotted is the mean and SE from three independent experiments. b) Cell cycle distribution at the indicated times for cells growing as in a. c) Characterization of cell cultures in a for Ki67 signal. In b) and c), experiments are reproduced 3 times and a representative one is shown.

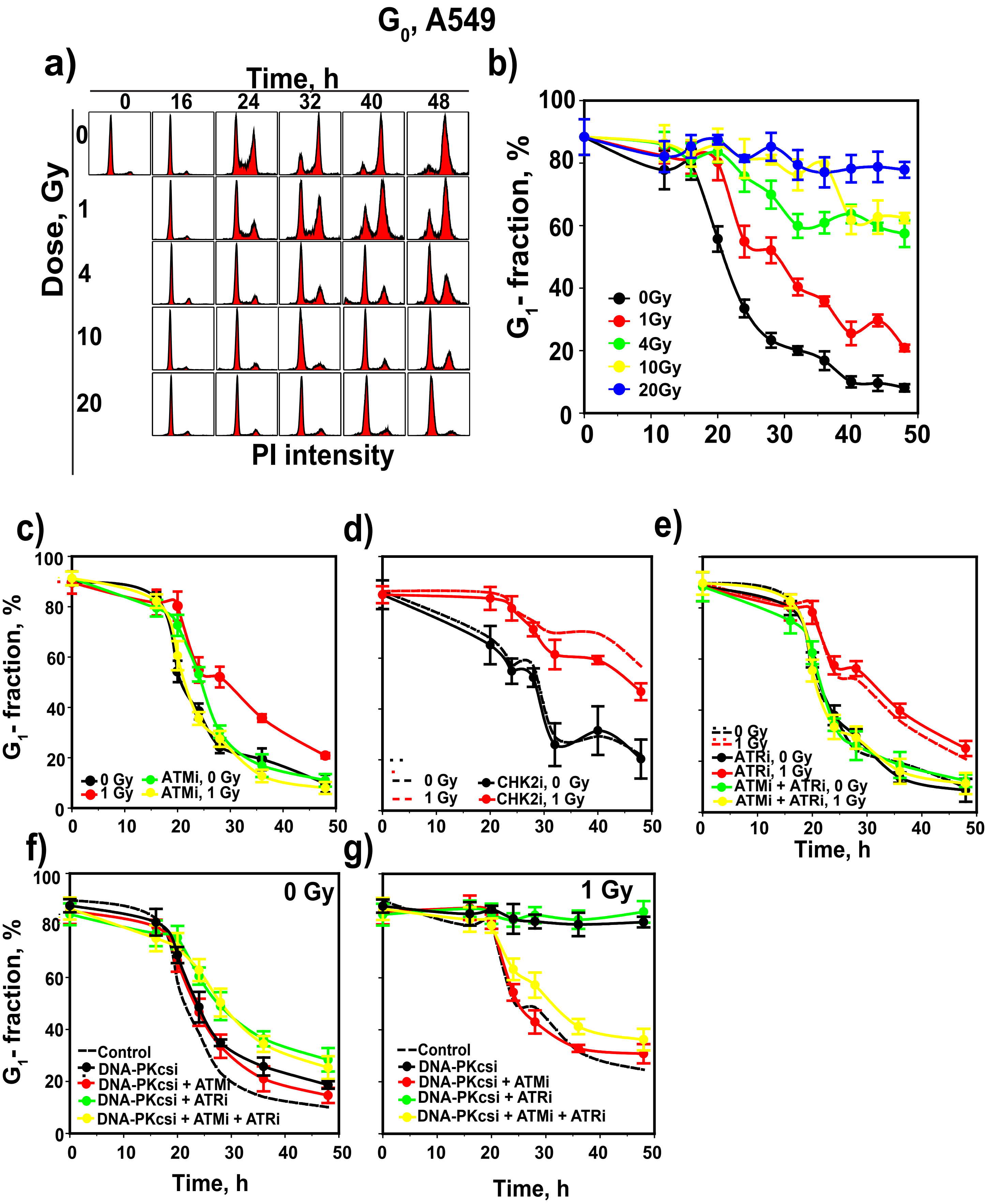

Li et al, Figure S6

Figure S6. IR-induced G1 checkpoint in G0 irradiated A549 cells. a) Representative flow cytometer histograms after irradiation and transfer to fresh growth medium obtained from 3 independent experiments. b) As in Figure 3b for A549 cells exposed to different doses of IR. c) As in b for cells exposed to 0 or 1 Gy and treated with ATMi. d) As in c for cells treated with CHK2i. e) As in c for cells treated with combinations of ATMi and ATRi. f) As in c for unirradiated cells treated with the indicated combinations of DNA-PKcsi, ATMi and ATRi. g) As in f for cells exposed to 1 Gy. Broken lines show results from c without data point to avoid congestion. Plotted is the mean and SE from three independent experiments. See also Table S9 for more information.

G$_0$, A549

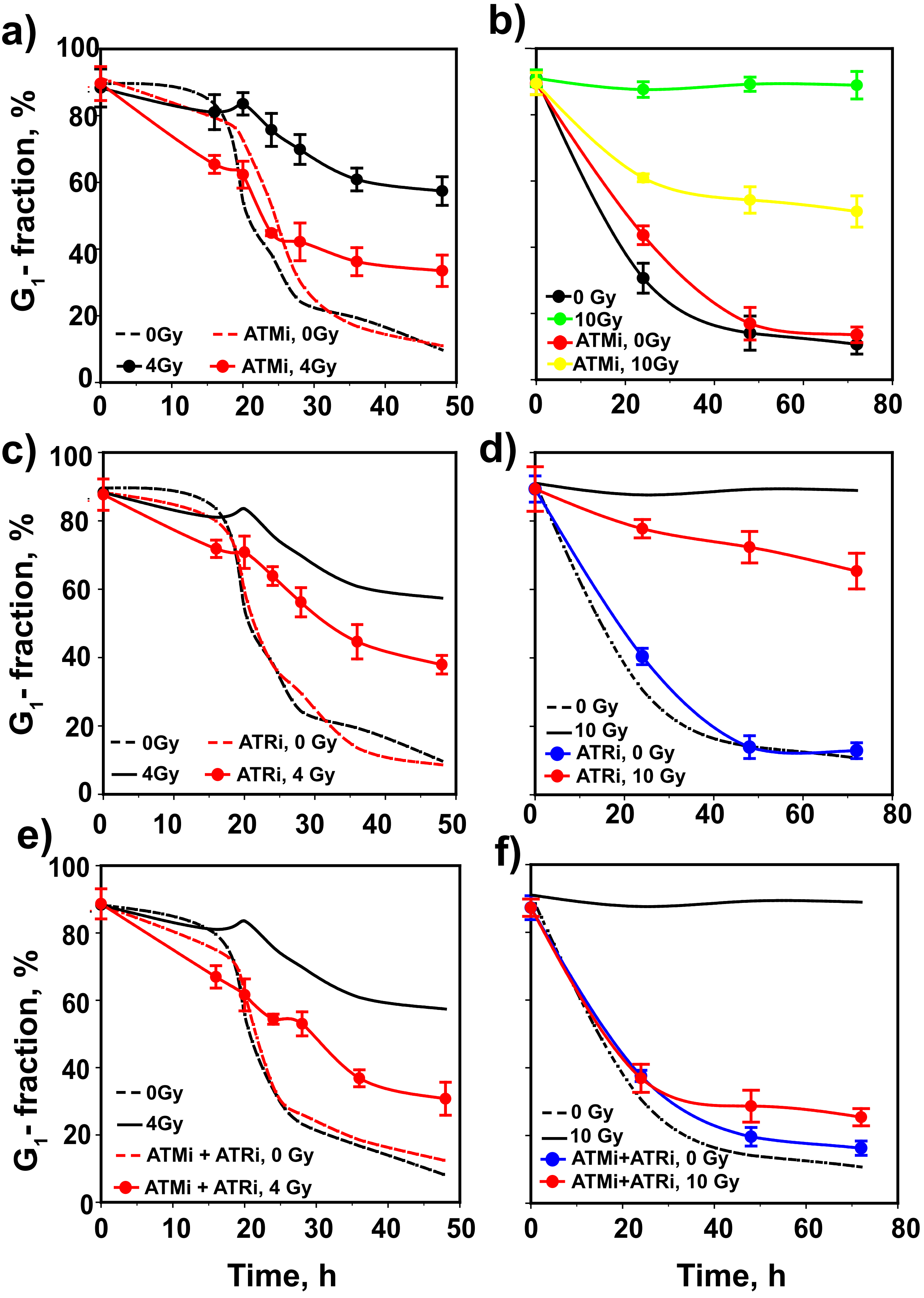

Figure S7 Li et al.

Figure S7. As in Figure 5 for A549 cells. Lines of both broken and solid show results from Figure S6 or S7b without data point to avoid congestion. See also Table S10 for more information.

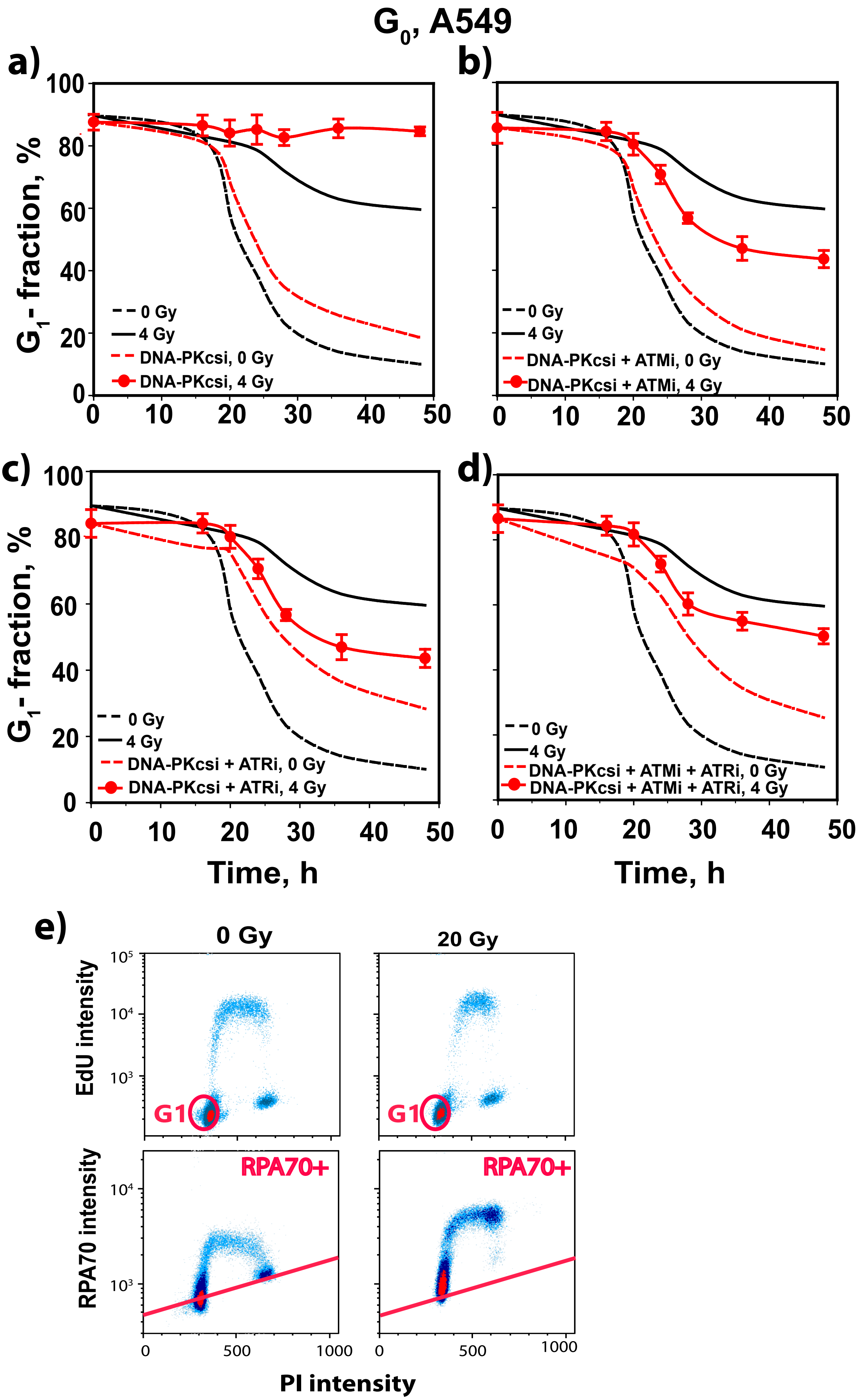

Figure S8, Li et al.

Figure S8. a)-d) As in Figure S4 for A549 cells. Lines of both broken and solid show results from Figure S6 or S7 without data point to avoid congestion. Plotted is the mean and SE from three independent experiments. See also Table S11 for more information. e) Example of the approach used to quantitate DNA-end-resection at DSBs by measuring RPA70 intensity in G1-phase of cells. The upper panel shows the gating of G1-phase cells using PI and EdU signal. The lower panel shows the gating used for the detection of RPA70 signal.

Table S1. Statistical analyses of Figure 1

Fig 1b	0Gy Vs 1Gy	1Gy Vs ATMi, 1Gy	1Gy Vs ATRi 1Gy
16h after IR	**	**	N. S
24h after IR	**	**	N. S

Fig1c	0Gy Vs 10Gy	ATMi, 0Gy Vs ATMi, 10Gy	10Gy Vs ATMi, 10Gy
36h after IR	**	**	**
48h after IR	**	**	**

Fig 1d	0Gy Vs 10Gy	10Gy Vs ATRi, 10Gy	10Gy Vs CHK1i, 10Gy	10Gy Vs CHK1-2i, 10Gy
36h after IR	**	*	**	**
48h after IR	**	N. S	*	*

Fig 1e	ATMi+ATRi, 0Gy Vs ATMi+ATRi, 10Gy	ATMi+CHK1i, 0Gy Vs ATMi+CHK1i,10Gy
36h after IR	N. S	N. S
48h after IR	N. S	N. S

Fig 1f	0Gy Vs DNA-PKcs, 0Gy	DNA-PKcsi, 0Gy Vs DNA-PKcs, 10Gy
36h after IR	**	**
48h after IR	**	**

Table S2. Statistical analyses of Figure 3

Fig 3b	0Gy Vs 1Gy	0Gy Vs 4 Gy	0Gy Vs 10Gy	0Gy Vs 20Gy
36h after IR	**	**	**	**
40h after IR	**	**	**	**
48h after IR	**	**	**	**

Table S3. Statistical analyses of Figure 4

Fig 4a	0Gy Vs 1Gy	ATMi, 0Gy Vs ATMi, 1Gy	1 Gy Vs ATMi, 1Gy
36h after IR	**	N. S	**
48h after IR	**	N. S	**

Fig 4b	1Gy Vs CHK2i2, 1Gy	1 Gy Vs CHK2i1, 1Gy
36h after IR	N. S	N. S
48h after IR	N. S	N. S

Fig 4c	1Gy Vs ATRi, 1Gy	1Gy Vs CHK1-2i, 1Gy	1Gy Vs CHK1i, 1Gy
36h after IR	**	N. S	N. S
48h after IR	**	N. S	N. S

Fig 4f	0Gy Vs 10Gy	10Gy Vs CHK2i2, 10Gy	10 Gy Vs CHK2i1, 10Gy
36h after IR	**	N. S	N. S
48h after IR	**	N. S	N. S

Table S4. Statistical analyses of Figure 5

Fig 5a	0Gy Vs 4Gy	ATMi, 0Gy Vs ATMi, 4Gy	4 Gy Vs ATMi, 4Gy
36h after IR	**	*	**
48h after IR	**	**	**

Fig 5b	0Gy Vs 10Gy	ATMi, 0Gy Vs ATMi, 10Gy	10 Gy Vs ATMi, 10Gy
36h after IR	**	**	**
48h after IR	**	**	**

Fig 5c	ATRi, 0Gy Vs ATRi, 4Gy	4 Gy Vs ATRi, 4Gy
36h after IR	**	**
48h after IR	**	**

Fig 5d	0Gy Vs 10Gy	10Gy Vs ATRi, 10Gy	10Gy Vs CHK1i, 10Gy	10Gy Vs CHK1-2i, 10Gy
36h after IR	**	**	**	**
48h after IR	**	**	**	**

Fig 5e	ATMi+ATRi 0Gy Vs ATMi+ATRi, 4Gy	4 Gy Vs ATMi+ATRi, 4Gy
36h after IR	N. S	**
48h after IR	N. S	**

Fig 5f	ATMi+ATRi, 0Gy Vs ATMi+ATRi,10Gy	ATMi+CHK1i, 0Gy Vs ATMi+CHK1i,10Gy	10Gy Vs ATMi+ATRi, 10Gy	10Gy Vs ATMi+CHK1i,10Gy
36h after IR	N. S	N. S	**	**
48h after IR	N. S	N. S	**	**

Table S5. Statistical analyses of Figure 6b

	ATMi	ATRi	ATMi+ATRi	DNA-PKcsi	ATMi+DNA -PKcsi	ATRi+DNA-PKcsi	ATMi+ATRi+ DNA-PKcsi
**Un. tr**	*	**	**	N. S	**	*	**

Table S6. Statistical analyses of Figure S1

Fig S1a	0Gy Vs 1Gy	ATMi, 0Gy Vs ATMi, 1Gy	1 Gy Vs ATMi, 1Gy
18h after IR	**	N. S	**
24h after IR	**	N. S	**

Fig S1b	0Gy Vs 1Gy	1Gy Vs CHK2i2, 1Gy	1 Gy Vs CHK2i1, 1Gy
8h after IR	**	N. S	N. S
16h after IR	**	N. S	N. S

Table S7. Statistical analyses of Figure S2

Fig S2a	ATRi, 0Gy Vs ATRi, 1Gy	ATMi+ATRi, 0Gy Vs ATMi+ATRi,1Gy	1Gy Vs ATRi, 1Gy	1Gy Vs ATMi+ATRi,1Gy
18h after IR	**	N. S	N. S	**
24h after IR	**	N. S	N. S	**

Fig S2b	0Gy Vs 1Gy	CHK1i, 0Gy Vs CHK1i,1Gy	CHK1-2i, 0Gy Vs CHK1-2i, 1Gy
18h after IR	**	N. S	N. S
24h after IR	**	N. S	N. S

Fig S2e	0Gy Vs 10Gy	CHK2i2, 0Gy Vs CHK2i2,10Gy	CHK2i1, 0Gy Vs CHK2i1, 10Gy
8h after IR	**	**	**
16h after IR	**	**	**

Table S8. Statistical analyses of Figure S4

Fig S4a	0Gy Vs 4Gy	DNA-PKcsi, 0Gy Vs DNA-PKcsi, 4Gy	4Gy Vs DNA-PKcsi, 4Gy
36h after IR	**	**	*
48h after IR	**	**	*

Fig S4b	DNA-PKcsi+ATMi, 0Gy Vs DNA-PKcsi+ATMi, 4Gy	4Gy Vs DNA-PKcsi+ATMi, 4Gy
36h after IR	**	**
48h after IR	**	**

Fig S4c	DNA-PKcsi+ATRi, 0Gy Vs DNA-PKcsi+ATRi, 4Gy	4Gy Vs DNA-PKcsi+ATRi, 4Gy
36h after IR	**	*
48h after IR	**	N. S

Fig S4d	DNA-PKcsi+ATMi+ATRi, 0Gy Vs DNA-PKcsi+ATMi+ATRi, 4Gy	4Gy Vs DNA-PKcsi+ATMi+ATRi, 4Gy
36h after IR	N. S	**
48h after IR	*	**

Table S9. Statistical analyses of Figure S6

Fig S6b	0Gy Vs 1Gy	0Gy Vs 4Gy	0Gy Vs 10Gy	0Gy Vs 20Gy
40h after IR	**	**	**	**
44h after IR	**	**	**	**
48h after IR	**	**	**	**

Fig S6c	0Gy Vs 1Gy	ATMi, 0Gy Vs ATMi, 1Gy	1 Gy Vs ATMi, 1Gy
36h after IR	**	N. S	**
48h after IR	**	N. S	**

Fig S6d	0Gy Vs 1Gy	CHK2i, 0Gy Vs CHK2i, 1Gy	1 Gy Vs CHK2i, 1Gy
40h after IR	**	**	**
48h after IR	**	**	*

Fig S6e	ATRi, 0Gy Vs ATRi, 1Gy	ATMi+ATRi, 0Gy Vs ATMi+ATR, 1Gy	1 Gy Vs ATRi, 1Gy	1 Gy Vs ATMi+ATRi, 1Gy
36h after IR	**	N. S	N. S	**
48h after IR	**	N. S	N. S	**

Table S10. Statistical analyses of Figure S7

Fig S7a	0Gy Vs 4Gy	ATMi, 0Gy Vs ATMi, 4Gy	4 Gy Vs ATMi, 4Gy
36h after IR	**	N. S	**
48h after IR	**	N. S	**

Fig S7b	0Gy Vs 10Gy	ATMi, 0Gy Vs ATMi, 10Gy	10 Gy Vs ATMi, 10Gy
36h after IR	**	**	**
48h after IR	**	**	**

Fig S7c	ATRi, 0Gy Vs ATRi, 4Gy	4Gy Vs ATRi, 4Gy
36h after IR	**	*
48h after IR	**	**

Fig S7d	ATRi, 0Gy Vs ATRi, 10Gy	10 Gy Vs ATRi, 10Gy
36h after IR	**	*
48h after IR	**	*

Fig S7e	ATMi+ATRi, 0Gy Vs ATMi+ATRi, 4Gy	4Gy Vs ATMi+ATRi, 4Gy
36h after IR	**	**
48h after IR	**	**

Fig S7f	ATMi+ATRi, 0Gy Vs ATMi+ATRi, 10Gy	10Gy Vs ATMi+ATRi, 10Gy
36h after IR	N. S	**
48h after IR	N. S	**

Table S11. Statistical analyses of Figure S8

Fig S8a	DNA-Pkcsi, 0Gy Vs DNA-PKcsi, 4Gy	4Gy Vs DNA-PKcsi, 4Gy
36h after IR	**	**
48h after IR	**	**

Fig S8b	DNA-PKcsi+ATMi, 0Gy Vs DNA-PKcsi+ATMi, 4Gy	4Gy Vs DNA-PKcsi+ATMi, 4Gy
36h after IR	**	*
48h after IR	**	*

Fig S8c	DNA-PKcsi+ATRi, 0Gy Vs DNA-PKcsi+ATRi, 4Gy	4Gy Vs DNA-PKcsi+ATRi, 4Gy
36h after IR	N. S	*
48h after IR	*	**

Fig S8d	DNA-PKcsi+ATMi+ATRi, 0Gy Vs DNA-PKcsi+ATMi+ATRi, 4Gy	4Gy Vs DNA-PKcsi+ATMi+ATRi, 4Gy
36h after IR	**	N. S
48h after IR	**	*
